# Early differentiation patterning of mouse embryonic stem cells in response to variations in alginate substrate stiffness

**DOI:** 10.1186/1754-1611-7-9

**Published:** 2013-04-09

**Authors:** Joseph Candiello, Satish S Singh, Keith Task, Prashant N Kumta, Ipsita Banerjee

**Affiliations:** 1 Department of Bioengineering, University of Pittsburgh, Pittsburgh, PA, USA; 2 Department of Chemical Engineering, University of Pittsburgh, Pittsburgh, PA, USA; 3 Department of Engineering and Materials Science, University of Pittsburgh, Pittsburgh, PA, USA; 4 Center for Complex Engineered Multifunctional Materials, University of Pittsburgh, Pittsburgh, PA, USA; 5 McGowan Institute for Regenerative Medicine, University of Pittsburgh, Pittsburgh, PA, USA

**Keywords:** Diabetes, AFM, Pluripotent

## Abstract

**Background:**

Embryonic stem cells (ESCs) have been implicated to have tremendous impact in regenerative therapeutics of various diseases, including Type 1 Diabetes. Upon generation of functionally mature ESC derived islet-like cells, they need to be implanted into diabetic patients to restore the loss of islet activity. Encapsulation in alginate microcapsules is a promising route of implantation, which can protect the cells from the recipient’s immune system. While there has been a significant investigation into islet encapsulation over the past decade, the feasibility of encapsulation and differentiation of ESCs has been less explored. Research over the past few years has identified the cellular mechanical microenvironment to play a central role in phenotype commitment of stem cells. Therefore it will be important to design the encapsulation material to be supportive to cellular functionality and maturation.

**Results:**

This work investigated the effect of stiffness of alginate substrate on initial differentiation and phenotype commitment of murine ESCs. ESCs grown on alginate substrates tuned to similar biomechanical properties of native pancreatic tissue elicited both an enhanced and incrementally responsive differentiation towards endodermal lineage traits.

**Conclusions:**

The insight into these biophysical phenomena found in this study can be used along with other cues to enhance the differentiation of embryonic stem cells toward a specific lineage fate.

## Background

Embryonic stem cells derived from the blastocyst of the embryo in its early development stages are characterized by their pluripotency. This pluripotent nature reserves the cell’s ability to differentiate into any of the primary germ layer precursor cells: ectoderm, mesoderm, and endoderm. These multiple germ layers give rise to all of the various cell types in the body, a functionality that is different from adult stem cells. Adult stem cells, such as mesenchymal stem cells (MSCs), are more limited in the number of cell types they are able to differentiate into. Embryonic stem cells depend on specific cues or signals to acquire the eventual lineage or cellular fate. Specifically, external cues can be the major factor driving the differentiation to a desired cell type. These external cues can be broadly grouped into soluble chemical signals or physical cues dependent on the nature and type of the immediate microenvironment. Understanding the cues necessary to drive differentiation to a desired ultimate cellular phenotype is extremely important in designing artificial scaffolds for tissue engineering purposes. One such external physical cue is the directed mechanical force contributed by the surface over which the cells attach and proliferate. The mechanical characteristics of the surface provide the external mechanical stimuli which are translated to the cell through integrins, the cell-matrix adhesion molecules [1]. Changes in the type of and spatial presentation of adhesion sites of an artificial matrix have been shown to influence hMSC differentiation [2]. Alterations in the surface topography, such as introduction of wrinkles on the surface of a hydrogel, are also known to direct the fate of the MSC lineage [3]. Recent studies have demonstrated that the biomechanical properties of the substrate itself, specifically stiffness, directly influence the differentiation of the attached cells [1,4-8]. Engler et al. [6] in their pioneering work demonstrated that materials of similar mechanical attributes to those of native tissues associated with a cell type are most relevant for stem cell differentiation towards the corresponding phenotype.

This response to substrate and matrix stiffness has been well documented in mesenchymal stem cell lineage fates [1,5,7]. The stiffness ranges associated with driving mesenchymal cell fate have generally been in the kPa - MPa range, which is biologically relevant for cartilage and bone [6,7]. Also, the effects of variances in the mechanical environment’s stiffness has influenced differentiation in various other adult stem cell types, such as human dental follicle cells and muscle stem cells [4,8]. While, much work has focused on various types of these adult stem cells, the effect of material stiffness in directing embryonic stem cell fate is not yet well established. Previous reports investigating embryonic stem cells report that increasing the matrix stiffness over a wide range of material stiffness, from 0.041 MPa to 2.7 MPa has helped promote osteogenic markers in murine ESCs [9]. Additionally, substrate stiffnesses mimicking the stiffness of murine ESCs have been shown to promote proliferation, maintenance, and pluripotency of the ESCs compared to cells cultured on stiffer substrates [10]. The effect of substrate stiffness on modulating endoderm differentiation is less explored as yet, and is our primary interest in this article. Previous work from our group demonstrated the feasibility of promoting endoderm leaning differentiation on fibrin substrate by modulation of fibrin gelation conditions [11] which affect the fibrin microstructure. Due to the complex fibrin microstructure it is difficult to attribute the changes in differentiation directly to macroscopic stiffness (Task K, D'Amor A, Singh S, Candiello JE, Jaramillo M, Wagner WR, Kumta P, Banerjee I: Specific microstructural cues correlate with endoderm differentiation of mouse embryonic stem cells on fibrin gels as revealed by a systems level approach, submitted work). Therefore, in the current report, we have chosen an inert substrate, alginate, to investigate the effect of modulation of substrate stiffness on early differentiation patterning of mESCs.

The focus of our work herein is to restore the functionality of the pancreas, which can be compromised due to either loss of beta cell mass or reduced beta cell function from a variety of disease pathogenesis such as Type I Diabetes, Type II Diabetes, severe chronic pancreatitis, and pancreatic cancer [12-14]. Our primary interest is in developing an embryonic stem cell (ESC) based therapeutic approach for replacing the lost beta cell mass by functionally differentiated ESC derived beta cells. Since the pancreas arises from endoderm germ layer, it is important to understand how the endoderm commitment of embryonic stem cells is specifically affected by the various external cues of the environment, particularly, in this case the mechanical environment. In order to accomplish this it is important to choose a substrate that can allow the stiffness to be tailored to that of the native tissue associated with the target cell without also contributing to changes in chemical signaling. Calcium alginate has been successfully implemented for Islet cell encapsulation and transplantation [15-17]. It is also well established that by varying the amount of cross-linking cation or concentration of alginate, the stiffness can be controlled [18]. Alginate gels are also inert with regards to the cellular attachment, allowing any cellular signaling due to adhesion to be controlled by modulation of the amount of ligand protein in the gel. It has been demonstrated that alginate gels can be synthesized to exhibit a wide range of mechanical stiffness values varying from relatively soft (~100 Pa) to moderately strong (~10 kPa) [19].

The objective of this study is therefore to determine the sensitivity of the spontaneous differentiation of murine embryonic stem cells to minor or major perturbations in the Young’s modulus of the alginate gel substrate without altering the chemical signaling states due to focal adhesion sites. We were able to fabricate gels of low Young’s modulus while also subtly varying the stiffness over a range of an order of magnitude. In this study, we have been successful in spontaneously differentiating, i.e. employing a soluble chemical free signaling environment, ESCs on engineering alginate substrates while noting varying levels of changing marker expression for each germ layer. We also recognized a particularly remarkable sensitivity of endoderm markers to subtle changes in the substrate stiffness. This response of embryonic stem cell differentiation to alginate gels of low mechanical stiffness is reported for the first time to the best of our knowledge. Such findings could be extremely useful for a range of applications in tissue engineering including the regeneration of bioartificial pancreas along with possibly working in conjunction with directed chemical cues to enhance control of the differentiating embryonic stem cells.

## Results

In order to thoroughly understand the sensitivity of differentiation of mouse embryonic stem cells (ESD3) to the mechanical properties of the soft alginate gel substrates, we cultured the ES cells for five days under identical chemical but varying substrate conditions. The cells were allowed to spontaneously differentiate, free of soluble chemical signals, on the alginate substrates of stiffness range in the same order as that of the native pancreas. The stiffness of the native pancreatic tissue was first measured using AFM nano-indentation to determine the desired stiffness range to be reproduced by the synthesized alginate gels. The alginate gel properties were then meticulously tailored by calibrating the concentration of both alginic acid and the calcium ion crosslinker with, each gel type being subsequently further characterized by AFM nano-indentation to determine the stiffness. Each substrate contained equal amount of fibronectin to promote cell attachment. We took phase-contrast images of the cells in culture to understand the morphology of the ES-D3 cells as they differentiate and proliferate. Proliferation rate of the ES cells differentiating on the alginate substrate was measured using commercially available AlamarBlue assay. After the 5 day time period we investigated the phenotypic commitment of the cells through real time RT-PCR. The resulting changes in gene expression were tested for their correlation to changes in the alginate gel stiffness following a statistical modeling procedure. Finally, the protein expression of two genes found to be upregulated was verified through immuno-fluorescent microscopy.

### Characterization of native unfixed mouse pancreas

**Figure 1:**
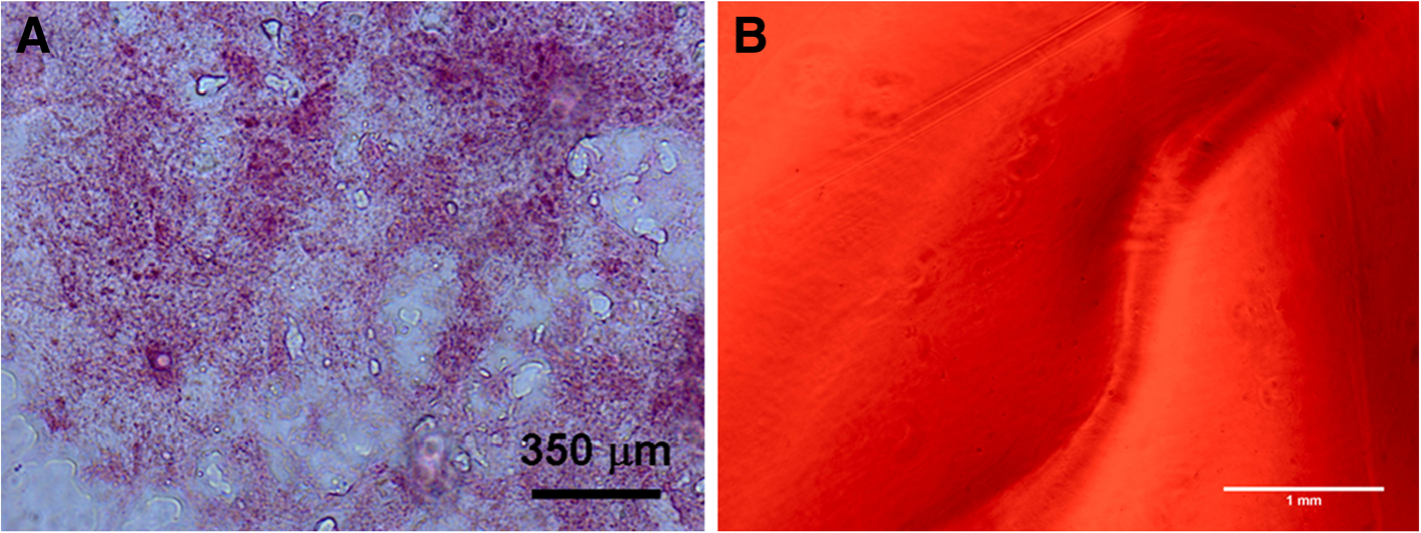
**H&E Staining of murine pancreas tissue (A) cryosectioned to 20 microns before fixation. **Staining was used to verify the tissue samples were intact, as subsequent sections were utilized for AFM stiffness measurements. Fluorescently tagged fibronectin gel (**B**) demonstrated that the fibronectin was homogeneously dispersed throughout the gel. A scratch can be seen in the image, which was necessary to provide a focusing point.

Measurements of unfixed native murine pancreas stiffness were taken on tissue samples cryosectioned and mounted on glass slides. The pancreas was sectioned to a thickness of 20 microns and the integrity was verified using H&E staining (Figure 1A). AFM force indentation measurements were then taken on n = 2 slide sections and approximately 50 force-indentation curves were taken on each at random locations. The Young’s modulus of elasticity for murine pancreas found was estimated as 1210 ± 77 Pa with individual measurements ranging from 618 Pa to 1407 Pa.

### Alginate gel characterization

Twelve alginate gel conditions, as indicated earlier, were synthesized by varying both the alginate and Ca crosslinking factor concentrations. The gel thickness was in the range of ~2 mm. The stiffness ranges of the various gels were measured by utilizing the AFM nano-indentation technique to determine the Young’s modulus of each alginate gel. At each of n = 3 randomly chosen locations on each gel, ~100 force-indentation cures were taken over a 10 × 10 grid with approximately 5 micron spacing between points. The Sneddon model was used to estimate the Young’s modulus at each point. The gel stiffness ranged from 242 ± 16 Pa for the softest gel to 1337 ± 27 Pa for the stiffest gel as detailed in Table 1. Since cells do not directly attach to the alginate gels, a constant concentration of fibronectin, 30 μg, was deposited on the gels one hour before plating the cells. We verified that the fibronectin was spread throughout the gel by tagging the protein with a FITC conjugation kit. Fluorescent images of the alginate gels demonstrated that the fibronectin was homogeneously dispersed throughout the gel (Figure 1B). A scratch was made in the gel to provide a focus point. Additionally, the Young’s modulus was measured before and after the coating of fibronectin was added. No noticeable difference in the bulk gel stiffness was observed.

**Table 1:** Alginate Gel Young’s modulus of elasticity

	**1× CL**	**2× CL**	**3× CL**
**6 mg/ml**	242 ± 16	330 ± 28	516 ± 39
**8 mg/ml**	389 ± 22	453 ± 12	659 ± 38
**10 mg/ml**	736 ± 54	854 ± 26	946 ± 15
**15 mg/ml**	1022 ± 103	1197 ± 72	1337 ± 27

Approximately 100 nanoindentation measurements were taken at n = 3 locations on each gel to determine the Young’s modulus of elasticity for each gel type.

### ESD3 cell morphology on alginate gel substrates

**Figure 2:**
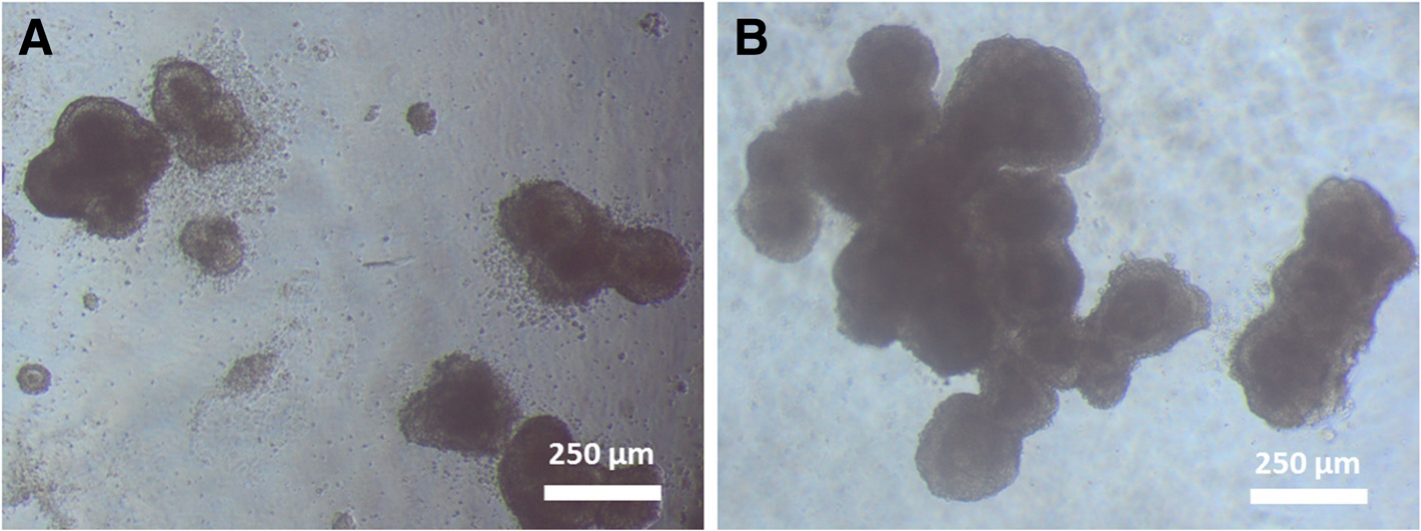
**Representative image of cell morphology of ES-D3 cells during spontaneous differentiation on alginate gels of varying Young’s modulus. **Cells on softer alginate gels (516 Pa, Figure 3A) were generally more numerous, but smaller after 3 days of spontaneous differentiation, while cells on stiffer substrates (1337 Pa, Figure 3B) formed larger clumps in a shorter time frame.

It was consistently observed in repeated experiments that some of the softer alginate gels were not robust enough to support the ES-D3 cells. A large portion of the cells sunk to the bottom of the well and attached to the underlying plastic of the 24-well plate. Hence these gel conditions: 6 mg/ml alginate concentration at 1× and 2× crosslinking concentrations along with 8 mg/ml alginate concentration at 1× crosslinking concentration were excluded from further analysis. For the gels that were able to support cell culture, phase contrast imaging of ES-D3 cells showed a clumped morphology (Figure 2). These clumps were not stagnant on the gel surface, but rather migrated on the gel surface, forming larger clumps as the cells proliferated and combined. Also, it was noted that the ESD3 cells generally formed larger clumps more immediately on stiffer gels (Figure 2B) in contrast to those on softer gel substrates (Figure 2A).

### ESD3 cell proliferation on alginate substrates

**Figure 3:**
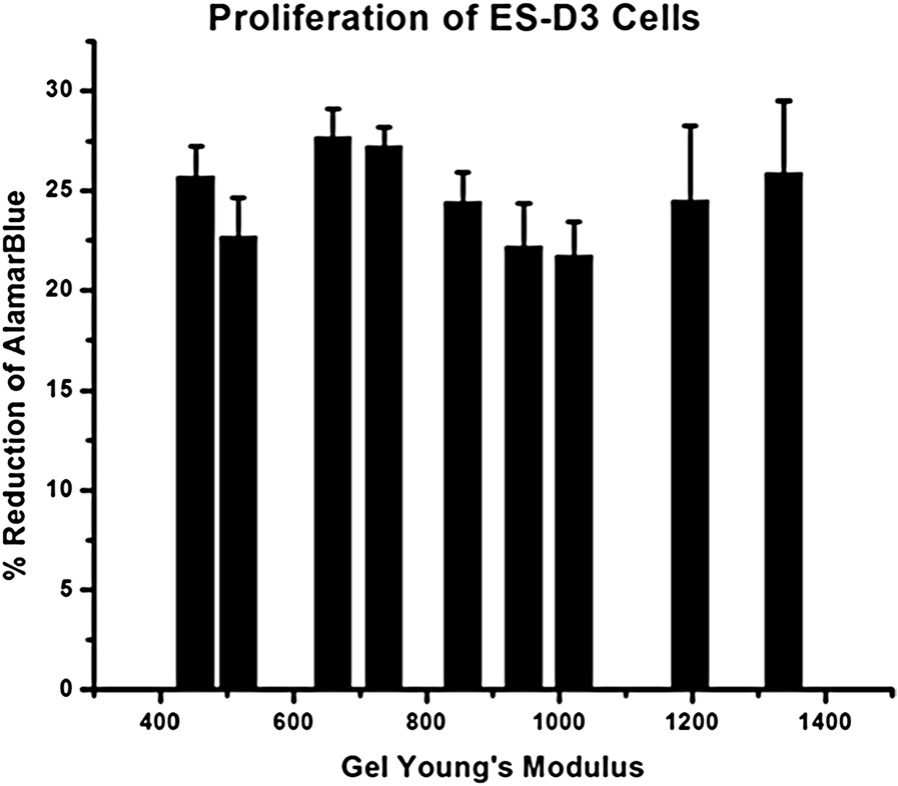
**Proliferation of ES-D3 was calculated by AlamarBlue Assay which relates the reduction of Alamar Blue due to cell metabolic activity to proliferation. **This study did not yield a relationship between proliferation and stiffness of the underlying substrate for the range of Alginate Young’s modulus studied.

ESD3 cell proliferation was studied at day 5 after plating cells on alginate substrates. Alamar blue assay showed that there was no appreciable trend in cell proliferation observed on the alginate gels in relation to variances in the stiffness of the alginate gels used in this study (Figure 3). For this assay, the proliferation of cells is directly related to the percentage reduction of Alamar Blue due to cell metabolic activity.

### Real time RT-PCR comparison of ES-D3 cells cultured on alginate gels of varying stiffness

**Figure 4:**
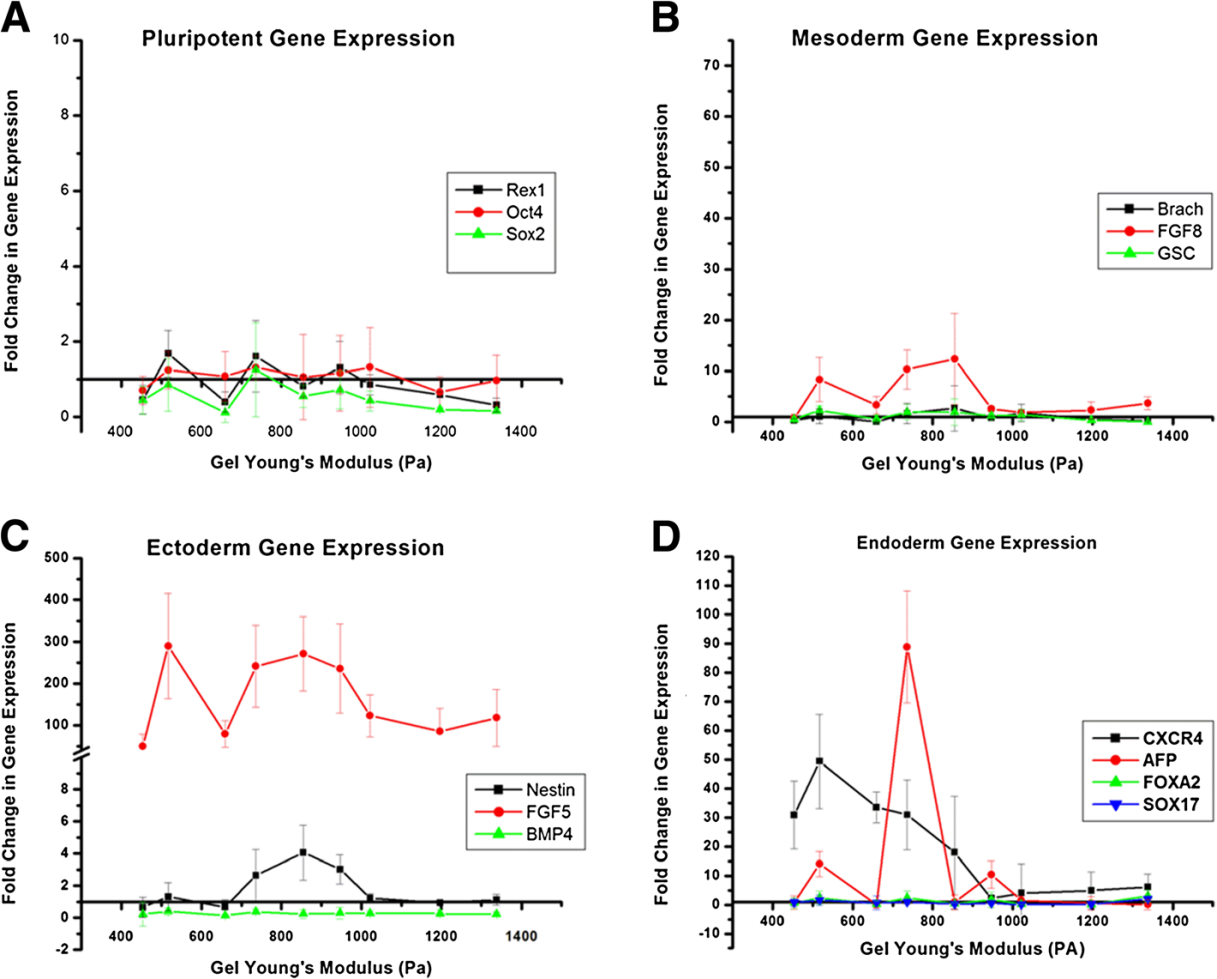
**Change in pluripotent (A), mesoderm (B), ectoderm (C), and endoderm (D) gene expression during spontaneous differentiation of ESD3 embryonic stem cells due to changes in substrate stiffness. **For pluripotent markers (Rex1, Oct4, and Sox2) there was either down regulation or no upregulation of gene expression after 5 days of differentiation. FGF8 (B, red line) was the only mesoderm marker that demonstrated any level of upregulation. The primitive ectoderm marker FGF5 (C, red line) was dramatically upregulated when compared to undifferentiated cells, however, this increase was notable for all substrate stiffnesses. The endoderm genes (D) CXCR4 and AFP demonstrated a strong upregulation in relation to the alginate substrates, while SOX17 and FOXA2 did not.

After allowing the ES-D3 cells to spontaneously differentiate on the various alginate substrates for 5 days in experimental media free of any additional soluble signaling factors, we analyzed the differentiated cell population for its phenotypic commitment. Our aim was to determine which germ layers, if any, were sensitive to the changes in mechanical properties of the underlying substrate. To accomplish this we chose representative markers for pluripotency and early markers for the three germ layers and analyzed it through real time RT-PCR. The pluripotency markers used for this study were *REX1*, *OCT4*, and *SOX2*. Figure 4A compares the relative change in these pluripotency markers across the nine alginate gels of varying Young’s modulus. For the most part we noted a downregulation of the pluripotency marker genes, and specifically very little sensitivity to the changes in gel stiffness. None of the markers demonstrated any significant upregulation as is expected with differentiation.

Next, in Figure 4C we analyzed the relative expression levels of three representative ectoderm genes: *Nestin*, *BMP4*, and *FGF5*. *BMP4* expression was downregulated across all alginate gel types, while *Nestin* demonstrated some low level upregulation of approximately 3 or 4 fold increase in the 700 to 1000 Pa range of alginate gel stiffness (black line, Figure 4C). *FGF5* (red line) showed very high upregulation across all gel types when compared to the undifferentiated ES-D3 cell, but its sensitivity to the varying gel stiffness was not appreciable. The sensitivity *FGF5* exhibited was however in the same range of the Young’s modulus of the alginate gel as *Nestin*. The sensitivity of mesoderm germ layer to varying alginate stiffness was analyzed through relative expression levels of *FGF8*, *GSC* and *Brachyury*. As illustrated in Figure 4B, RT-PCR gene expression analysis for *Brachyury* and *GSC* showed little to virtually no noticeable increase in expression when compared to the undifferentiated ES-D3 cells. There was also no sensitivity to gel stiffness. Of the mesoderm markers *FGF8* showed moderate upregulation of 8 to 12 fold, along with particular sensitivity between 650 and 950 Pa.

Gene expression analysis for endoderm germ layers captured multiple markers exhibiting both an increase in expression when compared to undifferentiated cells and a strong sensitivity to alginate gel stiffness, particularly in the lower ranges between 500 and 850 Pa (Figure 4C). *CXCR4* (black line) in particular showed a 30 to 50 fold increase in expression over this range. *AFP* (red line) exhibited up to a 90 fold increase in gene expression. The range of gel stiffness over which *AFP* and *CXCR4* showed particular sensitivity was also similar. However not all the endoderm markers exhibited this behavior, with *SOX17* and *FOXA2* being neither strongly upregulated nor sensitive to the gel stiffness.

### Immuno-fluorescence microscopy of mESCs

**Figure 5:**
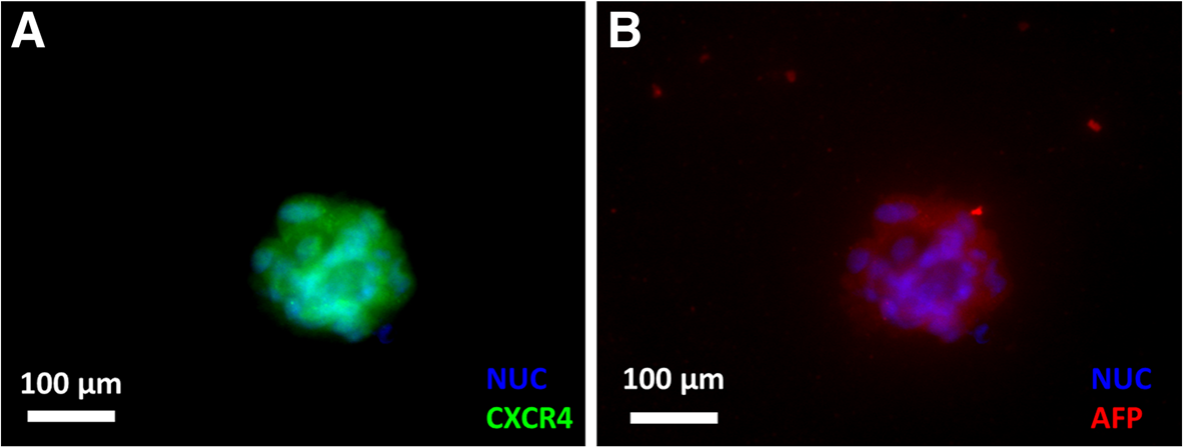
**Immunoflourescence Images of mESCs cultured on 10 mg/ml and 1× crosslinking alginate gels. **Cells expressed high levels of CXCR4 (**A**) and AFP (**B**).

For further verification of endoderm expression, we used immuno-flourescence imaging to verify protein expression of two of the higher upregulated genes found in the previous section. Immuno-staining of CXCR4 (Figure 5A) and AFP (Figure 5B) within a cell “clump” demonstrated clearly the presence of both proteins in the cells differentiated on the 10 mg/ml with 1X crosslinking alginate gel, selected as the representative gel condition.

### RT-PCR analysis of larger panel of endoderm specific gene

**Figure 6:**
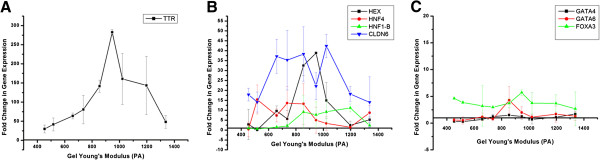
**Change in endoderm gene expression during spontaneous differentiation of ESD3 embryonic stem cells due to changes in substrate stiffness. **Endoderm marker TTR (**A**) demonstrated the highest amount of upregulation. Other endoderm markers demonstrated strong (**B**) upregulation also, while a small group of markers had very little to no upregulation (**C**).

After qualitatively noting an increase in endoderm specific gene expression in response to changes in alginate substrate stiffness, we conducted a more rigorous study into endodermal differentiation (Figure 6).

*TTR* (Figure 6A) demonstrated a particularly high increase in gene expression, with change in expression peaking around 275 fold. Figure 6B shows four endoderm markers (*HEX*, *CLDN6*, and *HNF4, HNF1-β*) that also demonstrated high upregulation in the range of 10 to 45 fold increase in expression. However not all the additional endoderm markers exhibited this behavior (Figure 6C), with *GATA4*, *GATA6*, and *FOXA3* not strongly upregulated. A summary of genes demonstrating strong upregulation is found in Table 2 (endoderm) and Table 3 (mesoderm, ectoderm, and pluripotency).

**Table 2:** Endoderm gene upregulation and correlation to gel stiffness

	**ENDODERM**											
	**TTR**	**HEX**	**GATA4**	**GATA6**	**FOXA3**	**HNFl-B**	**CLDN6**	**SOX17**	**AFP**	**HNF4**	**CXCR4**	**FOXA2**
Strong Upregulation	♦	♦			♦	♦	♦		♦	♦	♦	
Correlated to Stiffness	♦		♦			♦	♦				♦	

**Table 3:** Gene upregulation and correlation to gel stiffness

	**PLURIPOTENCY**			**MESODERM**			**ECTODERM**		
	**REX1**	**OCT4**	**SOX2**	**BRACH**	**FGF8**	**GSC**	**NESTIN**	**FGF5**	**BMP4**
Significant Upregulation					♦			♦	
Correlated to Stiffness						♦			

### Correlation of changes in gene expression to alginate gel stiffness

**Figure 7:**
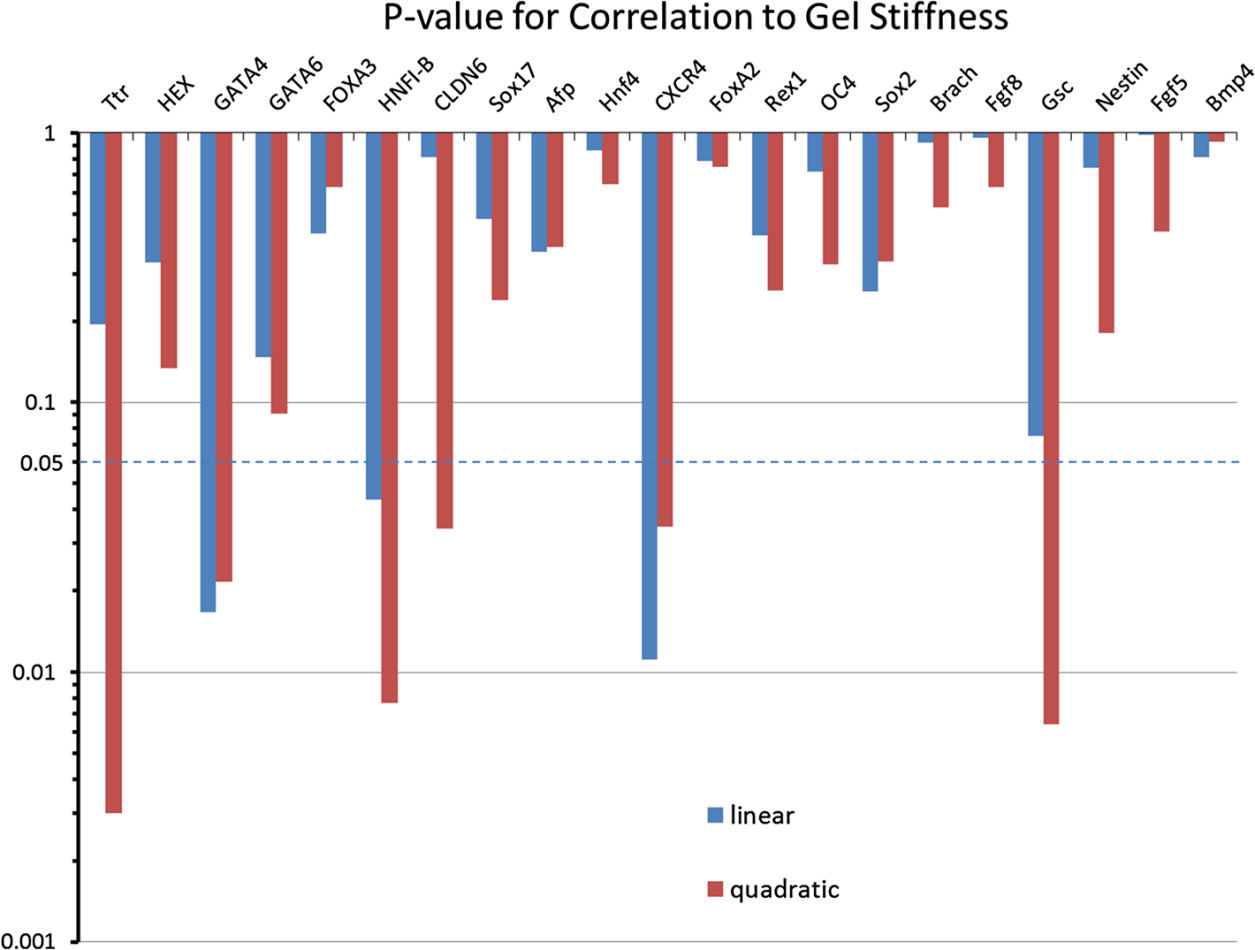
**P-value demonstrating the strength of correlation between the changes in gene expression and the changes in the alginate gel stiffness. **Values were determined for each gene marker investigated, and also for both linear and quadtratic relationships.

In addition to up-regulation, a qualitative inspection of the PCR data reveals that several of the endoderm genes are sensitive and strongly responsive to substrate stiffness. In particular, many genes show a bi-modal trend with a preferential up-regulation in a certain range of stiffness. However, a qualitative analysis is restrictive when describing this complex non-linear behavior, and a more quantitative approach is necessary to accurately compare the substrate responsiveness between genes. Therefore, we performed a more rigorous statistical correlation analysis of all of the analyzed genes for all germ layers, across all gel conditions. For each gene, we obtained a mathematical relationship of the gene expression responsiveness to gel condition by regressing the PCR data onto substrate stiffness. For each of these regressions, the p-value characterizing the significance of the mathematical relationship between the gene expression and gel stiffness was calculated (Figure 7). In this way, we were able to determine which genes show a strong correlation (p ≤ 0.05), either linear or quadratic, to the stiffness of the alginate gel. The genes *TTR*, *GATA4*, *HNF1*-*β*, *CLDN6*, *CXCR4*, and *GSC* were found to have gene expression strongly correlated to the variations in the substrates elastic properties. A summary of correlation is presented in Table 2 (endoderm) and Table 3 (mesoderm, ectoderm, and pluripotency) along with the summary of the up-regulation.

While multiple genes across the germ layers were up-regulated in response to the modulation of the substrate stiffness, our statistical correlation analysis reveals that not all these up-regulated genes are correlated to the substrate elastic modulus. For example, FGF8 and FGF5, representing mesoderm and ectoderm germ layer respectively, are both strongly up-regulated as shown in Figure 4B and 4C but correlate weakly with substrate stiffness. On the other hand, GSC, another mesoderm/ mesendoderm marker, shows a strong correlation to stiffness but is only weakly up-regulated. All the 5 genes which showed both high up-regulation and strong correlation were representative endoderm markers, as detailed in Table 2.

## Discussion

Stem cell lineage fate is influenced by both chemical and physical cues from the external environment. Recent work has also demonstrated that the stiffness of the material the cells are exposed to is one such physical cue important for influencing cell fate. The immediate goal of this study was therefore to demonstrate that modulations in the stiffness of a soft substrate over a relatively small range directly affect the gene expression of murine embryonic stem cells, and to specifically determine which germ layer lineages were most sensitive to changes in the substrate stiffness.

### Alginate gels as a suitable substrate for ESC differentiation

Alginate gels were chosen as a favorable substrate because of its inert nature, implying cells neither attach directly, nor do they receive any chemical cues directly from the substrate as the cells do not have any receptors for alginate chains [20]. Alginates are polysaccharides consisting of well-connected mannuronic and guluronic acids which are known to vary in amount and distribution [21]. Calcium, and other divalent cations, are known to crosslink the acid groups on multiple or alternate chains in aqueous media to form a two phase hydrogel [19,21].This allowed the stiffness of the substrate to be controlled, while maintaining a constant chemical signaling environment throughout the experimentation, by keeping a constant amount of fibronectin present. Alginate gels with a Young’s modulus range of 242 Pa to 1337 Pa were synthesized by varying the alginate concentration and the cross linking concentration (Table 1). The stiffness range was chosen to be in the range that the endoderm derived cells would be exposed to in the native tissue, since this is the main area of our research interest. Murine pancreatic tissue samples were measured by the same AFM nano-indentation technique, and determined to exhibit an average Young’s modulus of 1210 + 77 Pa with individual measurements ranging from 618 Pa to 1407 Pa.

In addition to our measurements, normal liver tissue (another endoderm derived organ) was observed to be of a similar, but slightly higher range of 2 to 4 kPa [22]. Cell interaction into the alginate gels were promoted through the introduction of a constant amount of fibronectin to the gel an hour before plating. Fibronectin diffusion throughout the gel was verified through fluorescently imaging gels with GFP tagged fibronectin (Figure 1B). In addition to having a constant amount of fibronectin in each gel, the soluble signal-free differentiation media used was the same throughout, which we postulated would isolate any difference in cell lineage fates to the variances in the substrate stiffness.

### ESC proliferation and morphological changes in response to soft substrate interaction

We studied the proliferation, morphology, and gene expression of ESD3 cells on substrates of relatively low Young’s modulus compared to many of the current studies of similar nature [9]. We were limited at the low end of substrate stiffness because of the gel stability itself. Gels corresponding to the low end of alginate concentration and calcium cross linking were unable to support cells for culture. The cells sunk to the bottom of the well-plate at these levels, spreading on the bottom of the well, and therefore were unsuitable for this study, as the underlying plastic of the tissue culture plate would also influence the cell behavior. The morphology of the cells on the alginate substrates was similar for all groups. Cells did not spread on the gels, but rather formed “tissue like” clumps. It is important to note that while the gels in our study did exhibit a 10 fold increase in stiffness, they were still observed to be in the soft range compared to many similar studies. It has been previously shown that cell morphology changes with substrate stiffness, spreading as the elasticity of the substrate increases [9,23]. It is likely that the alginate gels in this study did not reach a high enough level of stiffness for cells to show a more spread out morphology. While the cells did clump on our alginate substrate, it should be noted that the clumps did not stay in one place, but rather migrated on the surface of the substrate, eventually colliding with one another and forming larger clumps. Cell proliferation did not increase or decrease along with stiffness. Again, this may be due to the relatively small range of stiffnesses studied, as proliferation has been shown in other studies to be substrate stiffness dependent.

### ESC gene expression in response to soft substrate interaction

We studied the changes in gene expression for the differentiating ESCs using RT-PCR. Results demonstrated that unlike proliferation and morphology, gene expression was sensitive to changes in the substrate stiffness over this range of Young’s modulus. We demonstrated that pluripotent gene markers *OCT4*, *REX1*, and *SOX2* were either downregulated or showed no significant increase for all the synthesized gels (Figure 4A). This demonstrated that the cells did progress from their initial pluripotent state. There was also no particular sensitivity of the pluripotency markers to changes in the substrate elasticity (Table 3). Markers for mesoderm lineage showed varied response (Figure 4B). There was no upregulation in response to gel stiffness in the *GSC* and *Brachyury* mesoderm markers. There was however a 10 fold upregulation of the *FGF8* marker, which was sensitive over a range of approximately 700 to 950 Pa (Figure 4B). Since, *FGF8* is expressed during gastrulation and primitive streak formation on the mesoderm front, there is a possibility that a portion of the spontaneously differentiating ESD3 cell have undertaken characteristics of mesoderm lineage [24]. It is also possible that the morphological nature of the ESD3 cell clumping on the substrate surface may contribute to the upregulation of *FGF8*, as cells in low oxygen environments, such as in the center of a clump, overexpress *FGF8*[25]. Ectoderm specific markers revealed an interesting profile in gene regulation for the differentiating ESD3 cells (Figure 4C). While *BMP4* and *Nestin* showed either complete downregulation or only slight 2 fold increase when compared to undifferentiated ESD3 cells, the *FGF5* gene expression was greatly increased. In fact, *FGF5* showed the greatest increase of the markers studied. This approximate 100 to 200 fold increase in gene expression can possibly be attributed to the “gastrulation-like” state that the differentiating cells would be in. *FGF5* has been shown to be highly expressed in the mouse embryonic ectoderm, or epiblast, which gives rise to the three germ layers [26]. It has been shown to dramatically increase in mRNA expression at the beginning of gastrulation which was found in the cells that will specifically give rise to the definitive germ layers, and was then undetectable as germ layer formation and segregation is completed [26]. This may directly relate to the state of differentiation that the ESD3 cells were in at the time of RT-PCR analysis, and imply that the cells are in the stage at which lineage fates are determined. This may also be inferred based on the results demonstrating that while the *FGF5* levels were high for all gel conditions studied, there is no particular sensitivity to gel stiffness, similar to the previously discussed mRNA study. Initial Endoderm RT-PCR gene expression results showed, besides *FGF5*, the most dramatic increase in gene expression, and also the most sensitivity to changes in the substrate stiffness (Figure 4D). At day 5 of spontaneous differentiation there was no increase in gene expression for *SOX17* and *FOXA2*, however the endoderm gene markers *CXCR4* and *AFP* demonstrated an increase in gene and protein expression over the same range of substrate stiffness.

Additionally, a larger panel of endoderm genes was studied. Of these genes, there was no increase in expression for the *GATA4* and *GATA6* markers, while *FOXA3* demonstrated a slight increase (Figure 6C). Only *GATA4* demonstrated any correlation to substrate stiffness. *HEX*, *HNF4*, *CLDN6, TTR* and *HNF1*-*β* showed a noticeable increase in gene expression. It is possible that not all of the endoderm associated markers showed an increase in gene expression due to temporal expression profiles as is known and reported. For example, in murine embryonic stem cells spontaneously differentiating on PDMS, *FoxA2* expression did not increase until day 6 and beyond [9]. Nevertheless, we saw significant upregulation in 8 of the 12 endoderm markers along with a strong correlation to changes in the substrate stiffness in 5 of these cases (Table 2). By comparing this to the other early differentiation lineages which showed little upreguation and correlation to gel stiffness (Table 3), the importance of alginate and the ability to tailor the mechanical stiffness in the various ranges synthesized herein shows its dramatic influence on inducing early differentiation of ESD cells.

## Conclusion

In this study we demonstrated that chemically inert alginate gels are indeed a suitable substrate to study exclusively the influence of passive changes in the physical microenvironment on the embryonic stem cell differentiation. The properties of the synthesized alginate allow for controlled variation of the stiffness of the gels while maintaining a constant chemical signaling environment. At soft substrate stiffness we observed a spontaneous differentiation of pluripotent embryonic ESD3 murine stem cells towards lineage specific cell types. In particular our observation leads us to conclude that the endoderm lineage is both the most prevalent and also most sensitive to changes in gel stiffness, along with an increase in the gastrulation related gene marker. With these results showing the importance of the mechanical environment to cellular differentiation, it would be important to also consider the role of this factor for stem cell differentiation in any system. Also, understanding these physical cues would be critical when using them in conjunction with chemical signaling as an enhanced platform for guiding stem cell lineage fates.

## Materials and methods

### Embryonic stem cell culture

ES-D3cells used for this study were obtained commercially.ESD3 clonal mouse embryonic stem cells derived from blastocysts of 129S2/SvP as mice. The ES-D3 cells were maintained and manually cultured on 0.1% gelatin coated tissue culture flasks. The differentiation inhibiting media used for culture and passaging of ESD3 cells was KO DMEM (Invitrogen) supplemented with 15% knock-out serum replacement (KOSR) (Invitrogen), 1% penicillin-streptomycin (Pen-Strep), 4 mM L-glutamine (Cambrex), 1000 units/mL Leukemia inhibitory factor (Millipore), and 10 ug/mL Gentamicin. The media was replaced daily during culture and the cells were passaged every 3–4 days as they reached 75-80% confluency.

### Preparation of alginate gel substrates

A 15 mg/ml alginate stock solution was prepared by dissolving alginic acid (alginic acid sodium salt from brown algae, medium viscosity, Sigma) in de-ionized water. Solutions of 10 mg/ml, 8 mg/ml, and 6 mg/ml of alginate were then prepared from the stock solution by further dilution using de-ionized water. The bulk alginate hydrogels, of ~2-3 mm, were synthesized by a method previously described by Kuoet al. [18]. Briefly, 150 μL of alginate solution was thoroughly mixed with 75 μL of a suspension of calcium carbonate (CaCO_3_ powder, Fisher) in de-ionized water in order to ensure the homogeneous dispersion of CaCO_3_ particles within the alginate solution. To initiate cross-linking, 75 μL of a glucono-δ-lactone (GdL, Sigma) solution, whose molar concentration was kept at twice that of CaCO_3_, was added to the alginate-CaCO_3_ suspension. The mixtures were then stored at 4°C for at least twenty four hours in order to ensure complete gelation. Table 4 illustrates the concentrations of CaCO_3_ and GdL stock solutions that were used in order to vary the extent of cross-linking for all three alginate concentrations used in the study. For *in-vitro* studies, prior to seeding cells onto the hydrogels, 50 μL of a 0.6 mg/ml solution of fibronectin (FN, 1.0 mg/ml, Sigma) was evenly distributed onto the surfaces of the completely gelled scaffolds to ensure proper cell attachment.

**Table 4:** Calcium carbonate and glucono-δ-lactone concentrations used for various cross linking conditions employed in the synthesis of alginate hydrogels

	**CaCO**_**3 **_**conc.(mM)**	**GdL conc. (mM)**
**1×**	16	32
**2×**	32	64
**3×**	48	96

### Fibronectin gel distribution

In order to determine the nature of FN distribution on the surface, FN was first conjugated with FITC using a Fluorotag™ FITC conjugation kit (FITC1-1Kt, Sigma), and then dispersed on the surface of the alginate hydrogels, as was previously described by Guvendiren et al. [3]. The gels were then imaged using a fluorescence microscope (CKX41, Olympus).

### Preparation of pancreatic tissue for AFM measurement and histology

The entire murine pancreas was surgically removed for performing AFM measurements and conducting histology of the pancreatic tissue. The unfixed sample was then cryoprotected by soaking in 30% sucrose solution in PBS for 24 hours. Following this, the entire murine pancreas was then placed in a mold and covered in OCT cold embedding media. This was immediately frozen by exposure to dry ice and left at -80 degrees Celsius overnight. The OCT block containing the pancreas was cryosectioned to a thickness of 20 microns and mounted on SuperFrostPlus (Fisher Scientific) glass slides. Prior to stiffness measurements using the AFM, the sample was thawed for thirty minutes in PBS. All AFM measurements were conducted in PBS. For histological imaging of the cryosectioned murine pancreas, the slide mounted samples were first fixed in 4% glutaraldehyde and then prepared using hematoxylin/eosin stain to verify the integrity of the pancreas samples.

### AFM measurement of alginate gel and pancreatic tissue stiffness

AFM force indentation measurements were performed using the MFP-3D Atomic Force Microscope (Asylum Research, CA, USA), mounted on top of an Olympus IX-71 fluorescence microscope (Olympus, Tokyo, Japan). All the force measurements and analysis was conducted using the MFP3D software build (Asylum Research) built on the IgorPro 6 (Wavemetrics) platform. For all the measurements a glass silica sphere (radius 3.5 micron) was attached to the tip of a commercially available silicon-nitride (Si_3_N_4_) cantilever with a spring constant (k) of .238 N/m (Veeco). The spring constant of the Si_3_N_4_cantilever was calibrated using the thermal fluctuation method [27]. The stiffness of each of the alginate gels was then accordingly measured by micro-indentation with indentations made at randomly chosen locations considering approximately n = 100 force indentation curves [28-30]. The Sneddon model was used to determine the stiffness of the gels using the force indentation plots [28,29,31,32].Correlations of the indentation dimension with the force applied was conducted for a spherical tip model using the following equation relating the force (f) and the sample indentation size (δ):

(1) $$f=\frac{4}{3}\frac{E}{1-\upsilon^{2}}\sqrt{R}\delta^{\frac{3}{2}}$$

where E is the Young’s modulus, R is the radius of the spherical indenter, and νis the Poisson’s ratio. The sample indentation (δ) is calculated as follows:

(2) $$\delta =\left(z-z_{0}\right)-d$$

Where z_0_ is the initial indentation contact point, z is the position of the piezo-electric cantilever, and d is the cantilever deflection. The force-indentation curves were then fit to an indentation depth of 100–150 nm assuming the Poisson’s ratio to be ν = 0.5. The apparent Young’s Modulus was obtained by fitting the force-indentation curves to equations (1) and (2) with the initial deflection point and Young’s modulus (E) as the fitting parameters [33]. The curves were fit to small indentations (~100 nm) in comparison to the thickness of the samples.

### Experimental alginate culture conditions

To follow the differentiation effects of the substrate stiffness on the embryonic mouse ESD3 cells, the bulk alginate gels were formed at the bottom of each well of a 24-well plate. Fibronectin (0.6 mg/ml) was coated on top of each gel to allow for attachment of the cells. Approximately 60,000 cells were harvested and plated in the wells. The cells were cultured in an experimental media comprising DMEM containing 10% FBS, 1% P/S, and 1% NEAA, and then allowed to spontaneously differentiate, i.e. with no soluble or otherwise chemicals induced signaling, for 5 days. The media was replaced every other day.

### Cell morphology

The morphology of the differentiated stem cells was imaged over the course of the 5 day differentiation study. Phase contract images were then taken using an inverted Olympus light microscope employing a 10X lens. Images were taken immediately after the mESCs were plated on the alginate gels and intermittently over the entire 5 day period. Cells were kept under incubation temperature, and CO_2_ levels were maintained during imaging utilizing a Tokaihit incubation chamber mounted on the microscope stage. Prior to gel formation scratches were made at the bottom of the 24-well plate to ensure that the cells were located on the gel and had not migrated to the surface of the well plate.

### Alamar blue assay for cell proliferation

The relative proliferation between ES-D3 cells grown for 5 days on each alginate scaffold was measured by commercially available AlamarBlue Assay (AbDSerotec). Approximately 40,000 cells were plated in wells that had been coated with each alginate gel type and supplemented with DMEM (10% FBS, 1% P/S, and 1% NEAA). Wells of each experimental type were done in triplicate. Media was replaced at day 3. At the end of the 5^th^ day, AlamarBlue reagent was introduced to each well at a volume of 10% of total well volume, and allowed to incubate for 4 hours. After incubation, media containing the reduced AlamarBlue was removed from the alginate experimental wells and transferred to a 96-well plate to remove any interference from the gels. Absorbance readings at wavelengths of 570 nm and 600 nm were compared to the absorbance of negative control wells containing media and AlamarBlue only. The percent reduction of AlamarBlue was then determined using the following equation:

(3) PercentReduction=O2×A1-O1×A2R1×N2-R2×N1×100

Where: O1 = molar extinction coefficient (E) of oxidized AlamarBlue at 570 nmO2: E of oxidized alamarBlue at 600 nm; R1: E of reduced alamarBlue at 570 nm; R2: E of reduced alamarBlue at 600 nm; A1: absorbance of test wells at 570 nm; A2: absorbance of test wells at 600 nm; N1: absorbance of negative control well at 570 nm; N2: absorbance of negative control well at 600 nm

### RNA Isolation and reverse-transcription polymerase chain reaction (RT-PCR)

At day 5 the RNA was isolated from the cells of each alginate gel formulation using a Nucleospin RNA II kit (Macherer-Nagel). RNA concentration and purity were verified by measuring the absorbance via spectrophotometer. The extracted RNA was used to synthesize cDNA by ImProm II Reverse Transcriptase System (Promega). Gene expression analysis was performed on each experimental group using quantitative real time RT-PCR analysis. Multiple genes were targeted for each sample, focusing on gene markers for pluripotency (*REX1*, *OCT4*, *SOX2*) as well as the three germ layers: mesoderm (*Brachury*, *FGF8*, and *GSC*), endoderm (*SOX17*, *HNF4*, *CXCR4*, *FOXA2*, *TTR*, *AFP*, *CLDN6*, *HEX*, *HNF1*-B, *GATA4*, *GATA6*, and *FOXA3*), and ectoderm (*Nestin*, *FGF5*, *BMP4*) [11,34-46]. Primer sequences for each gene can be found in Table 5. Gene expression was then compared between the cells grown for 5 days on each alginate scaffold of differing stiffness and the initial undifferentiated cells (Eq. 4). The Ct for each marker was given by MxPro (Stratagene), and B-actin was used as the housekeeping gene to normalize between samples (Eq. 3). The relative fold change of gene expression between a sample and the undifferentiated control population is given by Equation 5.

(4) $${\mathrm{Ct}}_{\text{marker}\;\text{gene}}-{\mathrm{Ct}}_{\text{B-actin}}=\Delta \mathrm{Ct}$$

(5) $$\Delta {\mathrm{Ct}}_{\text{Cell}\;\mathrm{on}\;\text{Alginate}}-\Delta {\mathrm{Ct}}_{\text{Undifferentiatted}\;\text{ES-D}3}=\Delta \Delta \mathrm{Ct}$$

(6) $$\text{Relative}\;\text{fold}\;\text{change}\;\mathrm{in}\;\text{marker}\;\text{gene}=2^{-\Delta \Delta \mathrm{Ct}}$$

**Table 5:** Primer Sequences used for RT-PCR

**Gene**	**Sequence**	**Gene**	**Sequence**
Β-actin	R 5-tgg gag ggt gag gga ctt-3	**NEST**	R 5-ttc ccg tct gct ctg gtt-3
	L 5-cag cag ttg gtt gga gca-3		L 5-gga gga tgt ggt gga gga-3
FGF8	R 5-tga agg gcg ggtagt tga-3	**REX1**	R 5-tgg gag tca tcg ctt ggt-3
	L 5-acg gca aag gca agg act-3		L 5-aag gtc atc cac ggc aca-3
GSC	R 5-tcg ctt ctg tcg tct cga-3	**CXCR4**	R 5-atg acc agg atc acc aat cca-3
	L 5-gca ccg cac cat ctt ca-3		L 5-cgg gat gaa aac gtc cat tt-3
BMP4	R 5-cgc tcc gaa tgg cac ta-3	**TTR**	R 5-ggc aag atc ctg gtc ctc ct-3
	L 5-atc tgg tct ccg tcc ctg a-3		L 5-ttc aca gcc aac gac tct gg-3
FGF5	R 5-tag gca cag cag agg gat g-3	**FOXA3**	R 5-gcc cag tag gag cct ttg cc-3
	L 5-ttc aag cag tcc gag caa-3		L 5-cgg gcg agg tgt att ctc ca-3
OCT4	R 5-gct gat tgg cga tgt gag-3	**HEX**	R 5-tca gaa gag ctg tgg tta acc aa-3
	L 5-gga gaa gtg ggt gga gga a-3		L 5-agg ccg agt gtg aat cag ag-3
FOXA2	R 5-cgc cca cat agg atg aca tg-3	**CLDN**	R 5-tca caca ta att ctt ggt ggg a-3
			L 5-atg gcc tct act ggt ctg ca-3
	L 5-gtt aaa gta tgc tgg gag ccg -3		
HNF4	R 5-ccc tca gca cac ggt ttt-3	**HNF1B**	R 5-gga act ctg ata caa cac cag gct-3
	L 5-cat cgt caa gcc tcc ctc t-3		L 5-gcc tcc act cag gca cag agc-3
AFP	R 5-aac tgg aag ggt ggg aca-3	**GATA6**	R 5-ctc ttg gta gca cca gct ca-3
	L 5-ctc tgg cga tgg gtg ttt-3		L 5-gca atg cat gcg gtc tct ac-3
SOX17	R 5-aca cca cgg aggaaa tgg-3	**GATA4**	R 5-gag cag gag gca gac aag a-3
	L 5-atc caa cca gcc cac tga-3		L 5-gag caa ccg caa atc caa-3
BRACH	R 5-gcg agt ctg ggt gga tgt a-3	**SOX2**	R 5-ttg gat ggg att ggt ggt-3
	L 5-aag aac ggc agg agg atg-3		L 5-ctg gac tgc gaa ctg gag a-3

### Immuno-flourescene microscopy

Staining was performed on cells cultured for 5 days on a 10 mg/ml alginate gel formed with 1X concentration of cross linking. The staining protocol was performed according to recommendations from company. AFP goat polyclonal and CXCR4 rabbit polyclonal (Santa Cruz Biotechnologies) were used as primary antibodies. AlexaFlour 488 donkey anti-goat IgG (Invitrogen) and TexasRed donkey anti-rabbit IgG (Santa Cruz Biotechnologies) were used for secondary fluorescent labeling.

### Correlation of gene expression to changes in substrate stiffness

To quantitatively determine if a strong relationship was present between alginate stiffness and gene expression, the mathematical correlations between these two variables was assessed for significance. The first relationship to be identified was which genes, if any, show a strong linear dependence on the substrate stiffness. Therefore, the Pearson Correlation Coefficient between the response variable (expression data) and explanatory variable (alginate stiffness) was determined for each gene, as was the coefficient’s p-value. However, gene expression may exhibit a non-monotonic response towards substrate stiffness, a behavior which would not be captured by the Pearson Coefficient. Indeed, Figure 6A alludes to the possibility of quadratic response, indicating optimal stiffness conditions for the up-regulation of certain genes. To describe this behavior, a 2^nd^ order polynomial model was utilized:

(7) $$y=\beta_{o}+\beta_{1}x+\beta_{2}x^{2}$$

where y is the expression data and x is the stiffness. For each gene set, the expression data was regressed onto the stiffness data set (the latter being standardized by centering (by the mean) and scaling (by the standard deviation)) to estimate the unknown β parameters and form the estimated 2^nd^ order model. Significance levels of this model were determined by calculating the p-value of the overall regression model, which follows an F-distribution. For both the linear model and quadratic models, the fold change data was logarithmically transformed before analysis. Regression and statistical analyses were performed in Matlab (MATHWORKS).

To identify the genes which were significantly correlated to the substrate stiffness (both in the linear and quadratic models), an α value of 0.05 was chosen. Therefore, if the regression resulted in an overall regression p-value ≤ 0.05, then the correlation for that particular gene was considered significant.

### Statistics

Alginate gel stiffness measurements were repeated on n = 3 samples with approximately 100 measurements taken on each. ESD3 gene expression was conducted in n = 2 to n = 4 groups with duplicate samples measured for each. All results are reported with average and standard deviation values.

### Statement of ethical approval

All experiments and studies were carried out under IACUC protocol #1110987.

## Abbreviations

MSC: Mesenchymal stem cells; hMSC: Human mesenchymal stem cells; ESC: Embryonic stem cells; AFM: Atomic force microscopy; RT-PCR: Reverse transcription polymerase chain reaction; H&E: Hematoxylin and eosin stain; FITC: Fluorescein isothiocyanate; GdL: Glucono-d-lactose; CaCO3: Calcium carbonate; FN: Fibrinogen; Si3N4: Silicon nitride; DMEM: Dulbecco’s modified eagle medium; FBS: Fetal bovine serum; P/S: Penicillin/streptomycin; NEAA: Non-essential amino acids.

## Competing interests

The authors declare they have no competing interests.

## Authors’ contributions

JC carried out the mechanical property measurements and all biological experiments (culture, PCR, and proliferation) and drafted the manuscript. SS designed the alginate substrate and characterized all materials. KT performed all correlation to substrate stiffness calculation. PK and IB conceived of the study, participated in the design and coordination, and edited the manuscript. All authors read and approved the final manuscript.
